# Assessing patient-centered care in patients with chronic health conditions attending chiropractic practice: protocol for a mixed-methods study

**DOI:** 10.1186/s12998-016-0095-x

**Published:** 2016-05-09

**Authors:** Kent Jason Stuber, Mark Langweiler, Silvano Mior, Peter William McCarthy

**Affiliations:** Division of Graduate Education and Research, Canadian Memorial Chiropractic College, Toronto, Ontario Canada; Faculty of Life Sciences and Education, University of South Wales, Treforest, Wales; Canadian Memorial Chiropractic College, Toronto, Ontario Canada

**Keywords:** Chronic, Chiropractic, Patient-centered, Mixed methods, Study protocol, Chronic Care Model

## Abstract

**Background:**

The management of chronic health conditions increasingly requires an organized, coordinated, and patient-centered approach to care. The Chronic Care Model (CCM) has been adopted in primary care to improve care delivery for those with chronic health conditions. Chiropractors manage chronic health conditions; however, little is known if such care is patient-centered. The primary aim of this study is to determine to what extent chiropractic patients with chronic health conditions perceive their care is patient-centred. We will assess concordance with the CCM using the Patient Assessment of Chronic Illness Care (PACIC) survey in study patients. We will also explore perception of how patient-centered the care provided by chiropractors is for those with chronic health conditions according to patients and chiropractors.

**Methods/design:**

We will use a sequential mixed methods design with quantitative priority. In the quantitative component patients will complete a written questionnaire providing sociodemographic, health status, and health care interaction information, all of which will serve as the independent variables. Patients will also complete a modified version of the PACIC; the average overall score will be the dependent variable. In the qualitative component semi-structured interviews and focus groups with patients and chiropractors will be conducted. A pilot study will be conducted to determine if the modified PACIC will perform adequately in measuring concordance with the CCM for chiropractic care. Pilot testing will also allow for assessment of the interview and focus groups guides. Variables found to be significantly associated will be included in a multivariate linear regression analysis to identify significant predictors of the dependent variable. Qualitative data will be analyzed using an inductive thematic analysis to provide meaning to the quantitative results.

**Discussion:**

There is a paucity of research describing the extent to which chiropractic care for patients with chronic health conditions is concordant with the CCM. This study will examine this relationship and the perceptions and experiences of patients and chiropractors regarding how patient-centered chiropractic care is for these patients.

## Background

### Introduction

Chronic non-communicable health conditions are highly prevalent in Western society. The World Health Organization identifies four main chronic conditions: cardiovascular diseases, respiratory diseases, diabetes, and cancer [1]. Notwithstanding this, other chronic conditions including musculoskeletal conditions, e.g. arthritis, also have substantial impact on quality of life, mobility and independence, resulting in significant health care costs [2–4]. A large European study found that 19 % of respondents had moderate or severe chronic pain [5]. In that study among the most commonly cited body regions for chronic pain were back pain (lower back and unspecified back pain), knee pain, and head pain [5]. The most commonly cited causes of chronic pain were arthritis/osteoarthritis, herniated/deteriorating discs, and traumatic injury [5]. Population estimates of the prevalence of chronic conditions vary, often due to its differing definitions, but typically range between 25 and 50 % of the adult population [3, 6–8]. A commonly noted trend is the increasing prevalence of chronic conditions with advancing age [2, 4, 6–9]. Furthermore, as age increases, so too does the frequency of multiple chronic conditions within the same patient [6, 8]. Chronic conditions will become more prevalent as the demographics change and the population ages, with concomitant increases in health care demands and economic burdens [2, 9]. While there is still no consensus definition, typically a condition is defined as chronic if it has a prolonged duration and imposes a functional limitation on the patient that requires some form of health care intervention [9]. The length of the duration required for a disease to be classed as chronic can vary from 3 months to 1 year [9].

Chronic health conditions present unique challenges for patients, families, and health care professionals alike [10]. The long-term course of chronic conditions and their frequently changing impact on patients’ lives lead to a need for ongoing planning and decision-making with respect to treatment and self-management [10, 11]. As a result, Wagner et al. [11] developed the Chronic Care Model (CCM) to improve the delivery of care in patients with chronic health conditions [10]. The CCM is a multi-dimensional framework that has been widely adopted for managing chronic health conditions in a proactive, organized, patient-centered, and evidence-based manner, whether in large health care organizations or small clinics [10–12]. The CCM consists of six interrelated elements: health care system organization, links to community resources, self-management support, delivery system design, decision support, and clinical information systems [10]. Studies evaluating the implementation of the CCM suggest improved quality of care and outcomes in patients with chronic health conditions [12]. Preliminary evidence suggests that it may also be cost-effective in the long-term [12].

The CCM is patient-centered and emphasizes patient self-management in concert with organized care [13]. In 2001 the Institute of Medicine (IOM) identified patient-centered health care as one of its six specific aims for the improvement of health care [13]. The IOM defined patient-centered care as “care that is respectful of and responsive to individual patient preferences, needs, and values and ensuring that patient values guide all clinical decisions [13].” Patient-centered health care is multi-faceted; for example, the framework set forth by Mead and Bower [14] is composed of five dimensions: the biopsychosocial perspective, acknowledging the patient-as-person, sharing power and responsibility, creation of a therapeutic alliance, and acknowledging the doctor-as-person. Being patient-centered is a reasonable goal at both individual clinical and system-wide levels as it allows patients more input and control over their own health. There is increasing evidence that patient-centered approaches to care improve patient satisfaction, health behaviours and status for patients particularly when providers receive training in patient-centeredness and provide condition-specific educational materials and/or training for patients [15].

Chiropractic has been identified as being patient-centered [16–18]. Chiropractors predominantly see patients with musculoskeletal complaints; with spinal pain accounting for the majority of patients they see [19–22]. Chronic back and neck pain are common amongst these patients [23–27], who regularly present with other chronic conditions [20]. In Canada those with chronic back pain are three times more likely to see a chiropractor than those without chronic back pain [28]. Alarmingly, the rates of chronic back pain appear to be on the rise [23, 24] and spinal pain remains common as people age [26, 29].

Previous interview-based research with chiropractors has indicated that they consider patient-centeredness to be an important component of care [30, 31]. Surveys of other health professions have indicated that they perceive chiropractic care to be patient-centered [32, 33]. Increasingly authors are calling on chiropractors to be part of patient-centered collaborative care models [34–36] and trials are emerging that evaluate such models [37, 38]. However there are no published studies, to the authors’ knowledge, that address the degree to which chiropractic care for patients with chronic conditions is patient-centered. Given the emphasis placed on patient-centered care by patients and policy makers [13], it is imperative that the chiropractic profession quantifies the extent to which chiropractors practice in a patient-centered manner for those with chronic conditions. Such studies could lead to initiatives that promote changes in practitioner behavior, which would help align the profession more with the components of a patient-centered practice model, if necessary.

### Study aims

The primary aim of this study is to determine to what extent chiropractic patients with chronic health conditions perceive the care that they receive to be patient-centred. The primary objective of this study is to determine how patient-centered chiropractic care is for patients with chronic health conditions by assessing concordance with the Chronic Care Model as measured by the Patient Assessment of Chronic Illness Care (PACIC) [39–41].

A secondary aim of this study is to assess both the patients’ and chiropractors’ perception of how “patient-centered” the care provided by chiropractors is perceived to be for those with chronic health conditions. Perceptions and experiences will be explored using individual semi-structured interviews and focus groups guided by the framework of patient-centered care.

### Framework

Mead and Bower’s [14] model of patient-centered care will be used to frame our understanding of patient-centeredness. Strengths of this model include the equal emphasis placed on the patient and clinician and the importance of their relationship and communication, as well as the holistic approach to patients in how their life affects and is affected by their health problems. This model is suitable in a chiropractic setting given the holistic approach to care typically espoused by chiropractors [17] and the importance of a therapeutic alliance and communication between the chiropractor and patient [18].

## Methods

This project will begin with a pilot study. Both the pilot study and the main study will consist of two main components, a quantitative component followed by a qualitative component. The pilot study will be conducted to test the feasibility of the protocol and purposeful selection criteria and to develop the instruments and semi-structured interview questions. Upon completion of the pilot study any problems identified with the methods will be modified before initiating the main study. Such problems will be determined by asking participating patients, clinicians and clinical staff for their opinions. The investigators will keep a log of problems identified and determine appropriate solutions.

The study will employ a sequential mixed methods design, with a quantitative priority and a complementary qualitative approach [42–44]. This design has been chosen as it will allow the strengths of both qualitative and quantitative methods to be interwoven to provide an in-depth understanding of patients’ and chiropractors’ perspectives, perceptions, and experiences of patient-centered care in chiropractic. The qualitative data will be utilized to help inform and bring a deeper understanding of the quantitative data [42–45].

### Sampling

The pilot study will take place in two private chiropractic clinics in Calgary, Alberta, Canada. One is a large multidisciplinary sports injury clinic (including chiropractic, sports medicine, naturopathic medicine, physiotherapy, and massage therapy) with a chiropractor whose focus is primarily on musculoskeletal injuries and a smaller clinic that offers chiropractic and massage therapy and has more of a general chiropractic practice focus, meaning that there is no identifiable clinical specialization in areas such as sports injuries, pediatrics, geriatrics, rehabilitation, orthopedics, etc. The chiropractors at both clinics each have over 10 years of experience.

The main study will take place in fifteen chiropractic clinics across Canada. Different demographic areas will be represented by purposefully selecting private clinics located in municipalities with fewer than 50,000 residents, those between 100,000 and one million residents, and centres with more than one million residents. Private clinics will be recruited so that there is fair representation of both genders among clinicians, different levels of clinician experience, and types of practice (solo versus group versus interdisciplinary/multi-disciplinary), as well as chiropractic philosophical orientations. Clinics will represent at least five of the Canadian provinces to help ensure generalizability across Canada. Calgary, Alberta; Toronto, Ontario and Swift Current, Saskatchewan will be the sites for focus group interviews in the main study.

All patients will be recruited from the participating chiropractic clinics. Both chiropractors and office staff will be trained in patient recruitment methods by a Power Point presentation. This training will inform them of the inclusion and exclusion criteria in particular (described below) and provide advice of how to approach potential participants. A poster informing patients of the study will be placed in the reception/waiting area of all participating chiropractic clinics. Front desk staff at the respective clinics will ask consecutive patients if they are interested in participating in a research study. If so, they will be asked to read a Participant Information Sheet in the waiting room and consider their involvement. They will be able to ask the clinician questions about the study if necessary. After their visit, the staff will ask them if they are still interested in participating. After due consideration if they agree to participate they will then be asked to complete an Informed Consent form and accompanying questionnaire.

For the qualitative component, a subgroup of patients who provide their names, e-mail address or telephone numbers will be contacted by the Principal Investigator to arrange a time and place for the interview.

### Purposeful selection

For inclusion in the study all participants must be adults (over the age of 18 years) and able to read and speak English. Participating patients must have seen the same chiropractor at the respective clinic at least three times. This number of visits was selected so that patients would have sufficient familiarity with the chiropractic clinic and their approach to care to answer the questions posed to them. Participating patients must also have a chronic health condition. For the purposes of this study, a chronic health condition will be defined as any condition having a minimum 1 year duration affecting an organ system, including musculoskeletal, neurological, cardiovascular, etc. that has required health care treatment and/or resulted in some form of functional limitation or disability [9]. The chronic health condition does not necessarily have to be treated by a chiropractor to be considered eligible for inclusion. Participating chiropractors must be licensed to practice chiropractic in their province, and actively engaged in practice.

Potential participants will be excluded from the study if they are under the age of 18 years old, are unable to read and speak English, or are being treated for a new condition or re-aggravation of a previous condition. Chiropractors will be excluded from the study if they are not engaged in active practice or licensed in their respective provincial jurisdiction.

### Quantitative data collection

The questionnaire will ask sociodemographic and clinical information along with the modified version of the Patient Assessment of Chronic Illness Care (PACIC) [39–41]. Sociodemographic questions will include age, gender, ethnicity, and highest educational level. Clinical information will include the types of chronic health conditions that patients have, the types of health care providers that they see besides their chiropractor, the number of times they saw a chiropractor in the past 12 months, the length of time that they had been a patient at that clinic, and a subjective overall health rating.

The PACIC will be the primary outcome measure of this exploratory study. The PACIC is based upon the Chronic Care Model and measures the “receipt of patient-centered care” [39] and experience of care for those with chronic conditions [46]. The PACIC is widely used for assessing patients with a variety of chronic conditions and has been found to be reliable and valid [39–41, 46, 47]. Studies assessing individual subscale and overall PACIC Cronbach alpha scores have shown good internal consistency [39, 40, 47, 48]. Test-retest reliability for the subscales and overall PACIC score have also been found to be good [39, 41]. Glasgow et al. [39] worked with a large group of experts to develop the content validity of the PACIC and when tested the overall PACIC score had moderate to strong correlation with several convergent validity measures. Recent evidence supports construct validity of the PACIC as a measure of chronic illness care [40, 47, 48]. However, as Spicer and colleagues report (2012) [41], both confirmatory and exploratory factor analyses have provided mixed results, though they still recommend the widespread use of the PACIC. The PACIC has been validated for use in several different languages [47, 49, 50]. The PACIC is comprised of twenty questions including five subscales. The five subscales are:

i) Patient activation (three questions)
ii) Delivery system design/decision support (three questions)
iii) Goal setting/tailoring (five questions)
iv) Problem solving/contextual (four questions)
v) Follow-up/coordination (five questions).

Each question is scored using a five-point response scale where patients are asked to rate the frequency with which they receive a certain aspect of chronic care ranging from 1 = ‘almost never’ to 5 = ‘almost always’. The overall PACIC score is generated as an average by summing the scores for each question and dividing by the total number of questions (twenty). To generate subscale scores the average scores of the questions in each particular subscale are obtained. The overall mean PACIC score and subscore means each have a score between one and five, where higher scores are indicative of care that adheres more to the CCM and as such is more patient-centered [33, 39, 42, 51].

This will be the first study to use the PACIC in a chiropractic practice setting, although it has been used in a primary care setting for patients with a musculoskeletal condition, in particular with osteoarthritis [52]. Some items of the PACIC were modified for this study based on consultation with several practicing chiropractors, thus making it more appropriate for the chiropractic practice environment. The modified version replaces “health care team” and “physician” with “chiropractor” in the instructions and removes a sentence describing the possible composition of a health care team. One of the items from the original PACIC version had “medicines” replaced with “treatments” in the modified version. Another item from the original PACIC version replaced “doctor” and “nurse” with “chiropractor” in the modified version. In three additional items “health professionals” replaced “doctors”, “eye doctor” “specialist”, “dietitian”, “health educator”, and/or “counselor”. The modified version of the PACIC will not have any differences in terms of analysis methods when compared with the original PACIC.

Questionnaires will be distributed and collected by clinical staff onsite and stored securely upon completion. Completed questionnaires and consent forms will be placed into separate envelopes, sealed by the patient, and then collected by clinic staff and kept in a separate accordion-style file folder in a locked filing cabinet before being returned to the research team by secured courier. The Principal Investigator will store all questionnaires in a locked filing cabinet in a locked private office. Each questionnaire will be given a code known only to the Principal Investigator and maintained in a codebook that will be kept secured in a locked filing cabinet in a locked private office. The de-identified data in the questionnaires will be entered into a spreadsheet that is password protected on a computer that is further password protected.

For the pilot study, at each clinic forty consecutive willing patients with chronic health conditions will be enrolled in the quantitative component for a total of 80 participants [53, 54]. For the main study, participating clinics will continue to recruit subjects until the final sample size is reached. The final sample size determination for this quantitative component will be made after the pilot study [55]. However, Krucien et al. [47] identified 23 studies that have used the PACIC and sample sizes have ranged from 89 to 4108 subjects with an average of 1036 and median of 892.

### Quantitative data analysis

Quantitative analysis will include the reporting of descriptive statistics for sociodemographic, health status, and health care interaction variables, the means of the five different PACIC subscores, and the overall mean PACIC score with 95 % confidence intervals for those means [39, 51]. As per Jackson et al. [56] a mean minimum score of 3.5 on the different PACIC subscales and overall PACIC score will be set as a cutoff to indicate a high level of CCM concordance. Proportions of patients indicating high versus lower levels of CCM concordance will also be reported for the individual subscales and overall score. Bivariate analyses will be conducted by testing Pearson correlations for continuous variables and *t* tests for categorical variables. This will identify independent variables that are significantly associated with the dependent variable. The dependent variable in the model is the overall PACIC score, while sociodemographic characteristics (age, gender, ethnicity, educational level), health status (number of chronic health conditions, self-rated health), and health care interaction (number of health professionals interacted with and number of times seeing the chiropractor in the past year, length of time attending that particular clinic) will be tested as the independent variables. The majority of these independent variables have been evaluated in previous studies using the PACIC [47, 56–59]. Any independent variables found to be significantly associated with PACIC scores through the bivariate analyses will be included in a multivariate linear regression analysis to identify significant predictors of the overall PACIC score [60]. The coefficient of determination (R^2^), adjusted R^2^, F-test of overall significance and its p-value, and standardized (beta) and unstandardized (B) coefficients and their significance, will be determined. The model will also account for clustering around practice location [57].

### Qualitative data collection

The qualitative component will consist of three parts: (i) individual semi-structured patient interviews, (ii) individual semi-structured clinician interviews, and (iii) three focus group meetings that will include both patients and clinicians together. The Primary Investigator will conduct all interviews and focus group sessions, all of which will be approximately one to two hours in length and audio recorded with backup. If there are any occasions during the interviews where there is ambiguity or confusion surrounding something that a participant has said the interviewer will ensure that they ask for clarification and elaboration of the points being made. Another interviewing technique that could be used in such an event would be to reiterate what the participant said either verbatim or by paraphrasing and ask the participant to confirm or correct their understanding of the points being made. When the interviewer and participant are both satisfied that there is no longer any confusion, they will proceed to the next line of questioning. The qualitative components will take place after the analysis of the quantitative component with the focus groups following the individual interviews. The results of the quantitative analysis will form the basis of the interview guides developed for the qualitative components to aid with the understanding of the quantitative results. Similarly the quantitative and interview analyses will be used to help inform the focus group sessions.

i) Patient interviews - A subset of patients who complete the quantitative component and indicate interest in participating in individual interviews by providing their name and a contact phone number or e-mail address on their questionnaire will be asked to participate in individual interviews. The Primary Investigator will make attempts to have equal numbers of male and female subjects representing a range of ages interviewed. Between six and eight patients total will undergo the pilot patient individual interviews. Previous pilot studies of protocols involving semi-structured interviews have involved between two and 11 subjects [61, 62]. For the main study a sample size of 15 to 20 patient subjects is proposed, but recruitment will end once theoretical saturation has been achieved [63, 64]. A determination of theoretical saturation will be made as interview data are concurrently analyzed with interview data collection. The interviewer will meet the subjects at a neutral location at their convenience and conduct the interview in a private room with a single interviewer. Patients who are unable to attend the interview physically will be offered to be interviewed by telephone or videoconference.
  In the patient interviews, subjects will be asked open-ended questions regarding their perceptions and experiences of how patient-centered the care they receive is. An interview guide will be developed using the analysis of the quantitative component data and will also reflect Mead and Bower’s [14] framework of patient-centered care. These questions will be followed with probing questions to develop a deeper understanding of the patient’s perspective of the care they received.
ii) Clinician interviews - Chiropractors at the participating clinics will be asked to participate in individual semi-structured interviews. The purpose of collecting this information will be to present a different perspective of patient-centered care, that of the clinician and allow chiropractors to indicate how they perceive the care that they offer to be patient-centered for patients with chronic health conditions. This will allow for comparison with the information garnered in the patient interviews. The clinics will be recruited so that there is fair representation of both genders, different levels of clinician experience, locations (smaller versus larger centres and in different provinces), and types of practice (solo versus group versus interdisciplinary). In the pilot study four clinicians will be interviewed. For the main study, a sample size of 15 clinicians is proposed or until theoretical saturation is achieved [63, 64]. A determination of theoretical saturation will be made as interview data will be analyzed concurrently with interview data collection.
  The interviews will employ a similar structure to the patient interviews. If clinicians are unable to attend the interview physically, they will be offered the options of a telephone or videoconference interview. A separate interview guide will be developed for the clinicians based upon the analysis of the quantitative component data as well as the qualitative patient interview results and reflect the Mead and Bower framework [14]. These questions will be followed with probing questions to develop a deeper understanding of the clinician’s perspective of the care they provide and how it is patient- centered.
iii) Focus Groups – In the pilot, study patients from one of the selected clinics who are interested in participating in individual interviews will also be asked if they would like to take part in a pilot focus group interview as well. The pilot focus group interview will include one chiropractor and three-to-four patients as a “mini-focus group” [65]. In the main study focus group meetings will be conducted in three different municipalities (Swift Current, Saskatchewan; Calgary, Alberta; and Toronto, Ontario,). These municipalities are of different sizes ranging from fifteen thousand people to over five million people. One municipality (Swift Current) is in a rural setting, whereas suburban clinics will be used in another setting (Calgary), and the last (Toronto) is a large urban centre. Patients interested in participating in the individual interviews will also be asked to participate in the focus group sessions. Each of the three main study focus group meetings will include ten subjects consisting of two to three practicing chiropractors and seven or eight patients [65, 66]. It is desirable to have a greater number of patients due to possible perceived power differences in clinician-patient relationships.
  The purpose of the focus group sessions is to bring clinicians and patients together and have them engage in discussions about their perceptions and experiences of patient-centered care. The Primary Investigator will moderate the focus groups and meet all of the subjects at a neutral location at a time of their mutual convenience and conduct the interview in a private meeting room. For the focus group sessions, a separate interview guide will be developed. It will explore the findings of the quantitative and qualitative analysis from the individual interviews as well as both the Mead and Bower [14] and Chronic Care Models [10].

### Qualitative data analysis

The audio recordings from individual and focus group interviews will be transferred into password-protected audio digital files on a secure USB flash drive. All audio digital files will be copy protected. The secure USB flash drive will be stored in a locked filing cabinet in a locked private office. The audio digital files on the secure USB flash drive will be transcribed into a word processing document by a professional transcriptionist, saved only onto the secure USB flash drive and uploaded via the secure USB flash drive to a password-protected computer accessed only by the Principal Investigator in a locked private office. The documents will also be password protected. Participants in the interviews and focus groups will be given a code known only to the Principal Investigator and maintained in a codebook that will be kept secured in a locked filing cabinet in a locked private office. A professional transcriptionist will transcribe all interviews and focus group recordings verbatim with voice inflections and sounds described in parentheses. The primary investigator will double-check a random sample of 20 % of the transcripts against the audio recordings for accuracy.

Qualitative analysis of both interview components and the focus group sessions will consist of an inductive approach using “thematic analysis” methods [67]. As such emerging themes from the interview data will be developed through the analysis, not *a priori*. The Primary Investigator, along with an experienced social scientist (known hereafter as the reviewers), will conduct the analysis of the individual and focus group meeting data using qualitative data analysis software (NVivo). For the individual patient and clinician interviews the reviewers will code interviews separately and meet after coding the first five patient or clinician interviews respectively to ensure that they are generating similar codes. After every five subsequent interviews, the reviewers will meet to ensure that they are coding consistently. A third reviewer will resolve any disagreements. After fifteen patient or clinician interviews have been conducted the reviewers will each generate themes and subthemes emerging from the coded data [67]. These emerging themes will be discussed and refined between the two reviewers with any disagreements resolved by a third reviewer [67]. For the focus group interviews, the two reviewers will code the data after each focus group session and meet to ensure consistency in coding with a third reviewer resolving any disagreements. After all three focus group meetings have been completed and the interviews have been coded, the reviewers will generate emerging themes and subthemes. These will be discussed and refined by the reviewers with a third reviewer resolving any disagreements. Triangulation of sources will be done by comparing the themes and subthemes generated through the qualitative analysis of the practitioner and patient interviews and focus group interviews [45].

In studies employing qualitative methods a potential source of bias is that coming from the researchers themselves. To account for this the reviewers will ground themselves using self-reflection by keeping a journal throughout the process of data collection and analysis [68]. The journal will be used to write memos to track thoughts and consider how that may impact the way the study is conducted or analyzed.

### Ethical considerations

An information sheet and consent form will be presented to the participants by clinical front desk staff. Participants will be asked to review the information sheet and allowed to ask either the chiropractor or front desk staff questions about the project. If, after reading the study information sheet the patient is willing to volunteer for the study, the staff will witness their signature on the consent form. It is anticipated that most patients will choose whether or not to participate right away, but they will be allowed time to consider this and return at a later date (within 2 weeks) to complete the questionnaire if they so choose. A separate informed consent form will also be completed at the individual and focus group interviews.

All responses will be kept confidential. Patients will place their completed questionnaires in an envelope and seal them before giving them to clinic staff for secure storage. All records from the study will be kept private and appropriately secured. No personal information that may identify participants will be associated with participant responses in any reports. No publication that results from this study will contain identifiable information such as subject names. Manuscripts and presentations will be thoroughly reviewed and any possible identifying information will be removed.

During data entry each questionnaire will be given a code known only to the Principal Investigator for the purposes of tracking information. Any other members of the research team will only have access to de-identified data.

Those who choose to participate in the interviews or focus groups will be asked to provide their name and an e-mail address or telephone number so that the Primary Investigator alone may contact them to make suitable arrangements. Subjects will be asked not to provide their name or other identifying characteristics on the audio recording. Participants will be given a code known only to the Principal Investigator for use during the audio recording. Other members of the research team will only have access to de-identified data. The audio recordings will be transcribed by a professional transcriptionist to a password protected word processing file and uploaded to a password-protected computer accessed only by the Primary Investigator in a locked private office. At no point will the transcriptionist have access to any information that can identify the volunteers. Only the research team will have access to the data. All collected data will be retained for a period of 5 years. At that point, the Principal Investigator will shred all paper-based data and erase all data-containing digital and audio files from the audio recorders, secure USB flash drive, and computer.

Identified risks to both participating chiropractors and patients are deemed minimal and no physical risks are anticipated. The only identified disadvantage of taking part is due to the time involved in completing the questionnaire and/or the interview sessions. The greatest risk to participants is the disclosure of information provided to the research team in a manner in which the participant can be identified. Complete anonymity regarding focus group participation in this study cannot be guaranteed. Although focus group participants will be asked not to disclose anything that is discussed during the focus group, complete confidentiality cannot be assured. There is also the potential for patients and clinicians to know other individuals in the focus group sessions. The focus group sessions will take place in Toronto, Ontario; Swift Current Saskatchewan; and Calgary, Alberta and the use of only a small number of clinics in these settings is anticipated, consequently the potential for crossover of participants although possible, is deemed minimal. However, such an eventuality could reduce the anonymity of those sessions and may make some participants uncomfortable, therefore, attempts will be made to include only those patients who are not seeing the clinicians included in the study.

The only other identified risk in the individual and focus group sessions is that there is the small chance that an upsetting topic or memory may be discussed or cause some psychological distress. In the event that occurs, a referral to appropriate support services would be made available if further support is required. The topics discussed in the patient interviews should not be of a sensitive nature and, therefore it we do not expect to upset participants.

Participants who participate in the in-person qualitative interviews or focus group sessions will each receive a $50.00 gift card or pre-paid credit card in Canadian dollars to offset the costs of parking and transportation.

This study protocol has received ethical approval from the University of South Wales Faculty of Life Sciences and Education ethics subgroup (July 2015, approval LSE15KS36EO). A subsequent local approval was obtained from the Canadian Memorial Chiropractic College’s Research Ethics Board (October 2015, approval 1510X01). The researchers will not have access to patient files, personal details or diagnosis, other than that freely disclosed by the patient in the interviews or on the questionnaires.

## Discussion

The purpose of this study is to investigate how and to what extent chiropractic patients with chronic health conditions perceive the care that they receive to be patient-centred. To our knowledge this study will be the first to evaluate how patient-centered chiropractic care is for patients with chronic health conditions by assessing concordance with the CCM as measured by the Patient Assessment of Chronic Illness Care (PACIC).

Among the strengths of this study protocol is the use of mixed methods where both quantitative and qualitative data will be obtained including both individual patient and clinician interviews and focus group interviews that bring patients and clinicians together to assess perceptions and experiences of patient-centered care in chiropractic treatment. Another strength is use of a sequential mixed methods design, as the quantitative data will be used to help interpret the qualitative data. Finally collecting from a variety of different chiropractic clinical settings across Canada will help strengthen the generalizability of the results.

This study does not involve an intervention to increase patient-centeredness in chiropractic because of its exploratory design. However the results could potentially be used to inform future research to create interventions to address patient-centeredness in chiropractic.

## Abbreviations

CCM: Chronic Care Model
IOM: Institute of Medicine
PACIC: Patient Assessment of Chronic Illness Care
